# Piezoelectric Biopolymers: Advancements in Energy Harvesting and Biomedical Applications

**DOI:** 10.3390/polym16233314

**Published:** 2024-11-27

**Authors:** Menghan Xu, Yongxian Wen, Zhuqun Shi, Chuanxi Xiong, Fangju Zhu, Quanling Yang

**Affiliations:** 1 School of Materials Science and Engineering, Wuhan University of Technology, Wuhan 430070, China; xmh@whut.edu.cn (M.X.); wenyongxian@whut.edu.cn (Y.W.); cxiong@whut.edu.cn (C.X.); 2 School of Chemistry, Chemical Engineering and Life Sciences, Wuhan University of Technology, Wuhan 430070, China; zqshi2016@whut.edu.cn; 3 Zolia Quartz Stone Co., Ltd., Macheng 438300, China

**Keywords:** piezoelectric biopolymers, biodegradation, sustainable bioelectronics, energy harvesting, processing techniques

## Abstract

Biodegradable piezoelectric polymers have emerged as a hot research focus in bioelectronics, energy-harvesting systems, and biomedical applications, as well as in sustainable future development. Biopolymers possess plenty of features which make them promising candidates for next-generation electronic technologies, including biocompatibility, degradability, and flexibility. This review discusses piezoelectric biopolymers, focusing on the relationship between coupling mechanisms, material structures, and piezoelectric performance. Processing techniques such as annealing, mechanical drawing, and poling are introduced and further studied in terms of achieving high piezoelectric performance. This work reviews the strategies for enhancing piezoelectric properties via molecular engineering, nano structuring, and the incorporation of additives. Furthermore, the applications of these biopolymers in energy harvesting and biomedicine are provided, with a discussion of their potential in degradable bioelectronic devices. There are still challenges in optimizing piezoelectric performance and ensuring stability. Our research is expected to provide an understanding of these challenges and help to achieve a wider application of piezoelectric biopolymers.

## 1. Introduction

Piezoelectric materials can generate electrical responses (i.e., charge accumulation) when mechanical stress is applied to them [1,2,3]. Their applications include automotives, architecture, self-power technologies, and electronic products as well [4]. However, concerns about their environmental influence, such as lead toxicity from lead zirconate titanate (PZT) series, are rising, which hinders their viability [5]. Lead-free materials, such as perovskite barium titanate (BTO) and potassium sodium niobate (KNN), are not flexible and recyclable [6].

Numerous materials exhibit piezoelectric behavior, including the aforementioned BTO and PZT families [7]. Synthetic polymer materials, particularly PVDF, can also perform piezoelectricity [8,9]. The continuous exploration of both organic and inorganic materials with piezoelectric capabilities has broadened the applications of these materials [10]. The application of a high electric field induces nonlinear polarization, resulting in a characteristic hysteresis loop [11], like PVDF. Ferroelectricity indicates that the material can reverse its spontaneous polarization. Notably, all ferroelectric materials show piezoelectricity, but not all piezoelectric materials exhibit ferroelectricity [12]. PVDF exhibits piezoelectric and ferroelectric properties, yet poly(L-lactic acid) (PLLA) only shows piezoelectricity and does not demonstrate ferroelectric behavior. Indeed, PVDF possesses superior flexibility, biocompatibility, and processing, making it suitable for use in wearable, biocompatible, and biomedical electronic devices [13,14,15]. Yet the degradation of PVDF may induce toxic hydrofluoric acid (HF), which poses risks to human health [16].

Considering that bioelectronics are applied directly to human bodies, traditional piezoelectric materials raise significant concerns regarding biocompatibility [17,18,19]. Additionally, the biodegradability of materials is greatly desirable as it avoids the need for more procedures and improves both biocompatibility and environmental sustainability [20,21]. As a result, developments in biodegradable piezoelectric materials are greatly desirable for further applications in wearable and bioelectronic devices [22,23]. Natural biomaterials, such as protein-based polymers and polysaccharides, possess a fibrillar structure, which exhibits notable piezoelectric responses. Advancements in synthetic polymer materials also enable more and more piezoelectric materials via biochemical processes [24,25,26]. Natural and synthetic biomaterials can be degraded over time or safely reabsorbed by the human body. Strategies like molecular orientation adjustments and polarization tuning are explored to significantly enhance the piezoelectricity of these biodegradable materials, which may match or surpass that of their conventional piezoelectric counterparts, rendering these strategies promising in bioelectronics with biodegradable properties [27,28,29].

In this work, recent developments in biodegradable piezoelectric polymers are discussed. By systematically studying numerous biopolymers, their biodegradability, biocompatibility, and piezoelectricity are studied, with particular attention paid to environmentally friendly trend. We also explore the molecular mechanisms and their electromechanical coupling effects, which affect piezoelectric behavior. This study not only offers considerable investigation into the fundamental theory of piezoelectricity in these materials but also highlights their application and their enhanced piezoelectric performance. Moreover, we discuss the challenges to the application of these materials and propose future research directions that could further advance the development of biodegradable piezoelectric materials for biomedicine and a sustainable future. This work will promote the research into the next generation of sustainable piezoelectric materials, as well as developing more technologies for environmental protection and biocompatibility without decreasing their piezoelectric properties.

## 2. Piezoelectricity in Biopolymers

### 2.1. Biological Piezoelectric Polymers

**Figure 1:**
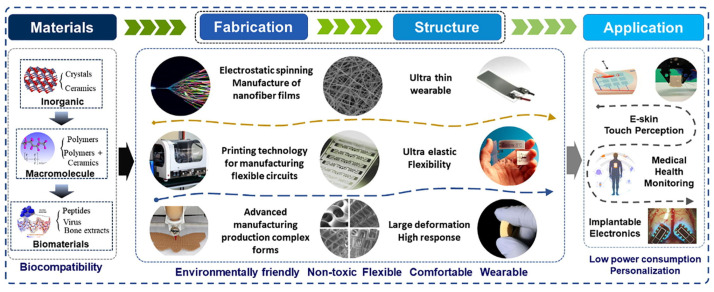
Piezoelectric materials for flexible and wearable electronics. Reprinted with permission from ref. [10]. Copyright 2021, ELSEVIER.

The increasing demand for sustainable devices and sensors is driving developments in smart electronics and flexible wearables, particularly in areas such as medical healthcare and human–machine interaction systems (see in Figure 1). These applications benefit from sensors that are not only flexible and stretchable but also breathable and highly sensitive, enabling the detection of environmental changes [30]. To advance them further, it is necessary to study the underlying mechanisms and theoretical models and explore more effective methods for preparing flexible piezoelectric materials. Biological polymers are characterized by high-order structures, low symmetry, and the lack of an inversion center, leading to linear electromechanical coupling [31]. Besides piezoelectric biopolymers like cellulose, chitin, and collagen, numerous other biological polymers have been developed into piezoelectric materials, further expanding their potential applications across various fields.

**Figure 2:**
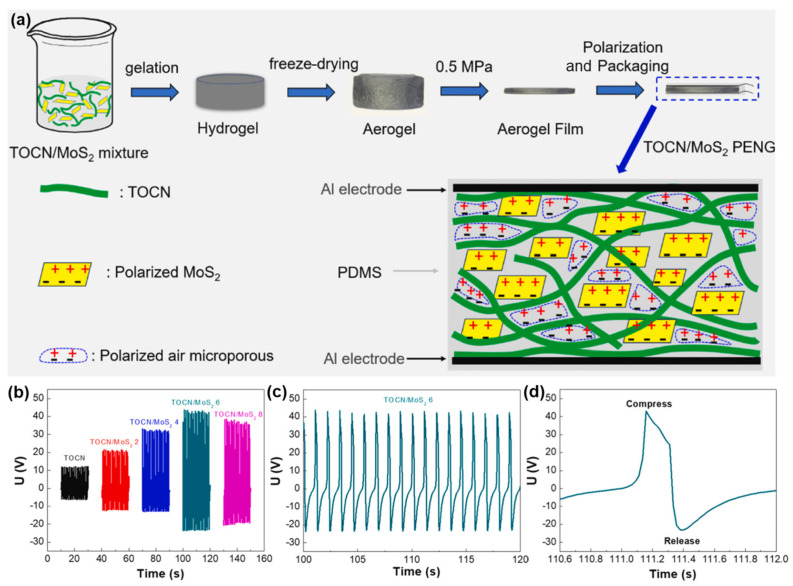
(**a**) Scheme of the preparation route of the TOCN/MoS_2_ aerogel films with different MoS_2_ contents. (**b**–**d**) Open-circuit voltage as a function of compression and release cycles for the porous TOCN and TOCN/MoS_2_ aerogel film piezoelectric nanogenerators. Reprinted with permission from ref. [36]. Copyright 2022, ELSEVIER.

**Figure 3:**
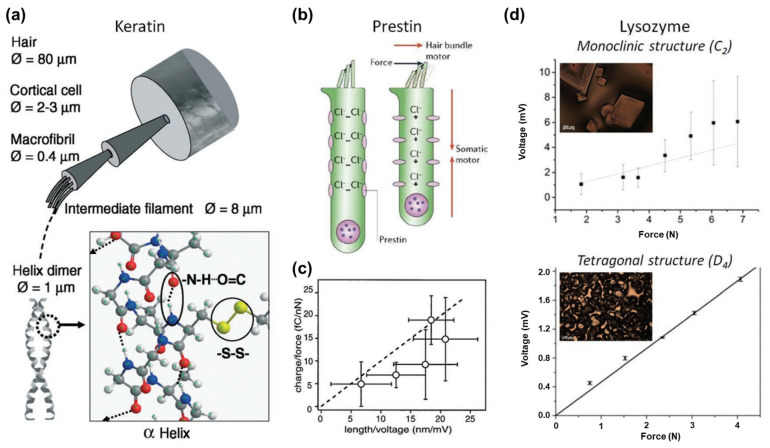
Piezoelectricity in keratin, prestin, and lysozyme. (**a**) The hierarchical structure of hair and keratin, as well as the piezoelectricity of keratin, are caused by an α-helical structure with hydrogen bonds. (**b**) Motility behavior of prestin. (**c**) Relationship between the charge transfer per applied force and the cell length per voltage. (**d**) The magnitude of the voltage generated increases with increasing force applied from monoclinic structured lysozyme (up) and tetragonal structured lysozyme (down). Inset: optical microscopy images of monoclinic and tetragonal aggregate films (below) of lysozymes. Reprinted with permission from ref. [41]. Copyright 2020, Wiley-CH.

Cellulose is a natural polymer composed of 1,4 glycosidic bonds. It forms fibril bundles in wood cells and was one of the first natural biomaterials with piezoelectric properties identified [32]. Fukada [33] quantified its piezoelectric response by determining that cellulose crystallites that were randomly distributed in the x-y plane were equally likely to be oriented in positive or negative directions along the z-axis, leading to low piezoelectric constants compared to quartz. Advances in technology enabled the extraction of cellulose from wood, revealing two polymorphic forms: Iα and Iβ. Iβ possesses a more stable monoclinic crystal structure [34]. Ultrathin cellulose nanocrystal (CNC) films, produced with the help of shear forces and an electric field, showed a high piezoelectric constant of 210 pC N^−1^ [35]. Other moderate piezoelectric responses have been reported in regenerated cellulose drawn under varying ratios. This piezoelectric effect is the most promising electromechanical coupling process for mechanical energy conversion and energy harvesting. Nevertheless, natural polymer-based piezoelectric materials still exhibit poor piezoelectric performance. As depicted in Figure 2, Yang et al. [36] developed flexible porous piezoelectric aerogel films based on 2,2,6,6-tetramethylpiperidine-1-oxyl radical (TEMPO)–oxidized cellulose nanofibrils (TOCNs) and MoS_2_ nanosheets. They possessed large specific surface areas and abundant mesopores. These films exhibited extremely good piezoelectric outputs, since a field strength of 20 MV m^−1^ was used to polarize the MoS_2_ nanosheets and air. When assembled to piezoelectric nanogenerators (PENGs), the TOCN/MoS_2_ aerogel PENG film that contained 6 wt.% of MoS_2_ exhibited the best performance output (see in Figure 3b), with an open circuit voltage of 42 V, a short-circuit current of 1.1 μA, a maximum area power density of 1.29 μW cm^−2^, and a maximum volume power density of 0.143 mW cm^−3^. These features enable it to be promising piezoelectric material for energy harvesting. Voltage outputs of 5 V and 12 V are generated by a 70 kg person during walking and running when a TOCN/MoS_2_ 6 PENG (diameter: 4 cm, thickness: 90 μm) is placed on the bottom of a sneaker to detect the energy from heel strikes. The fluctuations seen in the output signal can be attributed to the changing force during walking and running.

As natural carbohydrate materials, polysaccharides exhibit piezoelectric properties due to their asymmetric fibrillar structures. Chitin is an abundant polysaccharide and demonstrates piezoelectricity, which can be measured by piezoelectric force microscopy (PFM). There are three conformations in polysaccharides, i.e., α, β and γ [37]. The net polar response in β-chitin crystal has stronger uniaxial and piezoelectric effects. However, the net polarization of α-chitin along the electric field directions seems weak, which indicates marginal piezoelectricity in α-chitin [38]. Proteins such as collagen and silk are well studied for their piezoelectric effects. Collagen is a well-known protein-based material widely occurring in bones and connective tissues. Due to its triple-helix structure consisting of three twisted polypeptide chains, each of which has a characteristic repeating tripeptide sequence containing glycine, proline, and several amino acids, collagen shows electromechanical coupling during mechanical stress. Similarly, silk has three forms, i.e., random silk I, crystalline silk II, and threefold helical silk III. Among them, only silk II exhibits notable piezoelectricity, which is due to its highly crystalline β-sheet and orientation [39]. Silk I, with only random coils of protein polymers, forms an unstable amorphous structure, and silk III usually acts as a surfactant during silk processing. Kaplan et al. [40] found that samples with the highest drawn ratio of 2.7 showed the maximum shear piezoelectricity *d*_14_ of 1.5 pC N^−1^, increasing by more than two orders of magnitude; this was attributed to the increased crystallinity of β phase in silk II. This correlation between β-sheet in silk II and the ratio of drawing silk films indicates that mechanical drawing is effective in inducing piezoelectricity in silk and verifies that silk piezoelectricity is closely related to the combination of high β-sheet crystal and its orientation.

Keratin demonstrates piezoelectric properties due to its aligned α-helical dipoles; this shows the presence of piezoelectricity in hair and wool. The ordered arrangement of α-helical dipoles along the axis of keratin’s ordered structure was confirmed by studying the changes in piezoelectric behavior and crystal structure with temperature (see Figure 3a) [41,42]. Prestin, located in the outer hair cells (OHCs) of the mammalian cochlea, demonstrates considerable piezoelectric performance, contributing to high-frequency motility in cells, which assists in sensing sound vibrations. Although the precise mechanism of the piezoelectric effect in prestin is still being studied, its piezoelectric constant indicates its crucial role in biological electromechanical functions [43]. Experimental studies shown that OHCs contract and elongate in response to depolarization and hyperpolarization stimulation (see Figure 3b). The piezoelectric constant of prestin measures at about 20 mC N^−1^ for a 50 μm membrane (see Figure 3c) [44]. Lysozyme is an enzyme that is found in egg whites and various mammalian fluids, and it exhibits piezoelectric responses. Piezoelectric measurements of tetragonal and monoclinic crystals in lysozyme films revealed piezoelectric constants of 3.16 pC N^−1^ for the tetragonal phase and 0.94 pC N^−1^ for the monoclinic phase (Figure 3d). Although the response of lysozyme was not so impressive, which may be due to polarization cancelation in different domains, it still attracted attention for its potential use in biophysiological applications [45].

### 2.2. Synthetic Piezoelectric Biopolymers

In addition to natural biopolymers, many synthetic polymers that offer great flexibility in terms of tuning their properties, including piezoelectricity, have been produced in laboratories and industry [46]. Like most biological piezoelectric materials, synthetic polymers exhibit piezoelectricity by manipulating the conformation of their polymer chains and molecular structures. By emulating the mechanical strain-induced polarization of natural piezoelectric materials, novel biopolymers with the desired biodegradability can be designed and synthesized [47].

Polylactic acid (PLA) is a semi-crystalline polymer formed through the polymerization of lactic acid, which can be obtained from agriculture; lactic acid is also a product of human metabolism and is a good biological amphipath with the human body. PLA chains consist of ester bonds, which make them susceptible to hydrolytic degradation when exposed to a moist or microbial environment; they degrade into water and carbon dioxide [48]. Lactic acid exists in two enantiomers, L-lactic acid and D-lactic acid; poly (L-lactic acid) (PLLA) and poly (D-lactic acid) (PDLA), respectively, are formed during their polymerization. The dipole moment in PLLA originates from the electronegativity difference between the backbone atoms and functional groups, and it is also affected by structural asymmetries (i.e., chirality and helicity) [49]; its orientation in the carbon–oxygen double bonds (C=O) on the polymer backbone demonstrates strong shear piezoelectricity [50]. Dipoles in PLLA form by the orientation of polar groups, resulting in an asymmetrical arrangement of dipoles along the polymer chain. Although bending generates electrical responses, drawn PLLA exhibits a particularly strong shear piezoelectric effect because of the polymer’s molecular chain orientation, generating electric fields and enhancing the piezoelectric response.

**Figure 4:**
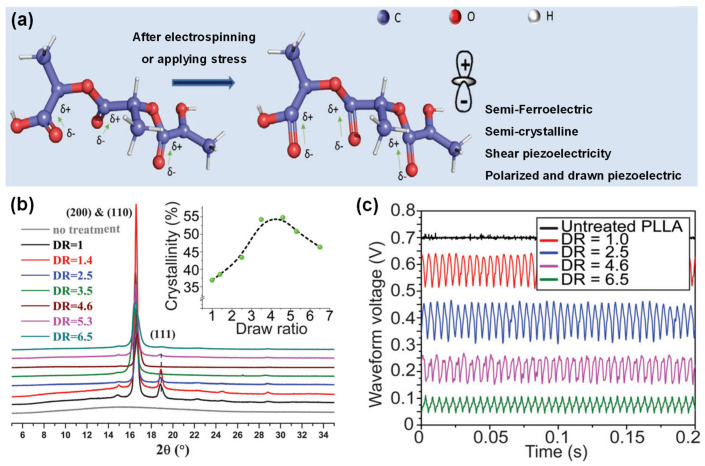
(**a**) Schematic of PLLA dipole alignment. Reprinted with permission from ref. [28]. Copyright 2018, Wiley-CH. (**b**) XRD pattern and (**c**) piezo-response comparison of PLLA films with different drawing ratios. Reprinted with permission from ref. [21]. Copyright 2018, National Academy of Sciences.

The molecular orientation and crystallinity of PLLA are strongly associated with its piezoelectricity; thus, its piezoelectric properties can be improved by controlling its molecular orientation and crystallinity. As mentioned, piezoelectricity in PLLA originated from the coordinated motion of dipoles within the polymer chains. Advances in processing and characterization techniques would enable a precise study of PLLA piezoelectricity at the micro- and nanoscale. Curry et al. [51] investigated the relationship between different drawing ratios (λ) and the piezoelectric performance of PLLA. As shown in Figure 4b, the pristine PLLA film had three crystal planes, (111), (200), and (110). With an increase in the drawing ratio, the intensity of the (111) peak dropped, which indicates a phase shift from the α-phase with 10_3_ helices to the β-phase with 3_1_ helices. The crystallinity of the PLLA was the strongest when the drawing ratio was 5 and then decreased with further stretching. Applying shear stress to PLLA films with different drawing ratios yielded the highest piezoelectric effect when λ ranged from 2.5 to 4.5, which was consistent with the trend of crystallinity. These results suggest that uniaxial drawing facilitates the alignment of polar C=O bonds, enhancing the piezoelectric effects.

Other typically widely used biodegradable polymers are polyhydroxyalkanoate (PHA) and its derivatives poly(3-hydroxybutyrate) (PHB) and poly(3-hydroxybutyrate-3-hydroxyvalerate) (PHBV), which exhibit twisted lamellar structures and varying orientations in formed films [52]. For instance, PHBV shows biocompatibility, biodegradability, and thermoplasticity, which are desired for biomedical applications. PHBV’s piezoelectric coefficient (1.3 pC N^−1^) closely resembles that of human bone, making it a promising biomaterial for biomedical applications such as tissue engineering [52]. PHBV is firstly degraded by an enzymatic mechanism, followed by hydrolysis and carbon dioxide release. In addition, combining the benefits of PHBV with hydroxyapatite (HA), PHBV-HA composites have shown potential for bone tissue engineering, suggesting their potential in regenerative medicine.

## 3. Effects of Processing on Piezoelectric Properties of Biopolymers

Film processing methods will greatly impact the piezoelectric performance of biopolymers [53]. Some processing techniques, like annealing, drawing, and poling, have proven useful and are usually employed individually or in combination. They could significantly impact the piezoelectric response.

### 3.1. Annealing

**Figure 5:**
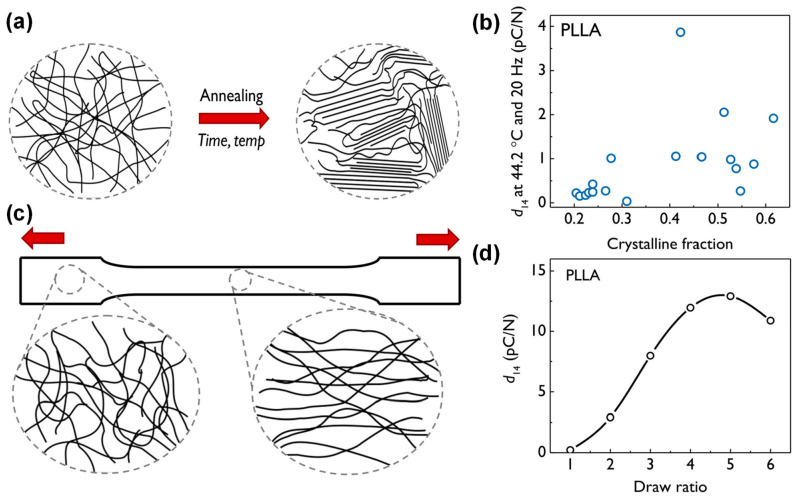
(**a**) Schematic representation of polymer crystallization as a result of annealing. (**b**) Influence of polymer crystalline fraction on the piezoelectric coefficient of PLLA. (**c**) Schematic illustration of the effect of drawing on a polymer sample. The material in the drawn region exhibits a significantly higher degree of orientation. The degree of orientation increases with the draw ratio. (**d**) Influence of the draw ratio on the piezoelectric coefficient of PLLA. A maximum value of *d*_14_ was observed at a draw ratio of ∼5. Data reproduced with permission from reference. Reprinted with permission from ref. [31]. Copyright 2021, Taylor & Francis.

Annealing (heat treatment or other type of temperature control) is often applied to adjust the crystallinity in biopolymers (see in Figure 5a) [31]. It is important to note that the center of symmetry remains, resulting in the polymer being isotropic after crystallization. Although the exact role of the crystalline and amorphous phases in piezoelectric properties is still unclear, annealing does appear to be effective in improving the electromechanical properties of piezoelectric biopolymers.

To study the effect of crystallinity on the piezoelectric response of PLLA, a formula for the piezoelectric constant *d*_14_ was proposed to factor in both the crystalline and amorphous phases. According to a reported study, the crystallinity of PLLA is usually between 30 and 50%, and the annealing temperature is usually set between 80 °C and 140 °C [54]. However, annealing is not always effective, especially when it affects the anisotropy of the material. Anisotropy at the molecular and structural levels is crucial for the performance of piezoelectricity at the macroscopic scale. Annealing-induced high crystallization may also produce anisotropic units, but the larger crystal structure may turn to isotropy, which will reduce the piezoelectric performance of the material. Therefore, annealing alone cannot ensure the realization of the ideal piezoelectric properties of the material, and it is usually necessary to combine it with other processing methods to further improve it.

### 3.2. Drawing

Drawing on biopolymer films usually starts at a higher temperature near the material’s melting point. As illustrated in Figure 5d [31], the processing direction aligns the polymer chains along the applied force, thus introducing the required anisotropy. Drawing is often exploited because it can align the amorphous and crystalline regions in the polymer matrix, resulting in piezoelectricity. The degree of piezoelectricity is usually related to the draw ratio, which is the ratio of the stretched length to the original length. In PLLA, the piezoelectric constant *d*_14_ increases with the draw ratio, reaching a peak at a draw ratio of about 5–6.

Generally, the α and α’ phases are common in PLLA structure, but drawing can induce a large amount of β-phase in PLLA, which is considered to be necessary for piezoelectricity. β-phase and piezoelectricity are not directly the result of the drawing mechanism, but the presence of the β-phase in PLLA is important for piezoelectric behavior. Piezoelectricity has been observed in PLLA with a draw ratio of 2, while the β-phase is usually formed at higher values (4 and above).

### 3.3. Poling

Poling can align the dipoles of materials by applying an external electric field [55]. This is particularly useful for ferroelectric materials, because these materials exhibit spontaneous polarization that can settle under a sufficient electric field. In polycrystalline ferroelectrics, polarization tends to be randomly oriented, but poling helps orient these dipoles uniformly, enabling piezoelectric behaviors [56].

In some cases, non-ferroelectric materials can also be polarized to induce dipole moments and surface charges, leading to the introduction of piezoelectricity. Poling can be performed by connecting the electrodes directly to the surface of the material or by directing the corona discharge near a sharp electrode [57]. The poling process is usually carried out while cooling the materials to the Curie temperature to ensure that the spontaneous polarization is oriented in one direction upon formation [58].

## 4. Methods to Enhance the Piezoelectric Response in Biopolymers

Many methods have been proposed to improve piezoelectric performance [59]; these include additives, fillers, modifications in processing techniques, and nanostructuring, and they are generally applicable to different biopolymers.

### 4.1. Additives and Fillers

Incorporating additives or fillers is a common approach to enhancing piezoelectric behavior [60]. This includes introducing materials with higher piezoelectric constants and using additives that are able to modify the structure of the polymer [61,62]. Since they are piezoelectric materials, PZT, BTO, TiO_2_, and ZnO particles are frequently used as additives [63]; these materials usually possess piezoelectric constants that are several orders of magnitude higher than those of many biopolymers [64]. They are typically added at the nano- or microscale to form piezoelectric composites with biopolymers. A critical consideration is to maintain anisotropy during fabrication, as this greatly affects the resulting piezoelectricity [65,66]. Additives without piezoelectricity can also be used, provided they have proved effective in modifying phase transitions and the polymer structure. Copolymers of poly(methyl methacrylate) and poly(butyl acrylate) have been shown to have selective positions in the crystalline regions of PLLA, enhancing chain orientation and the piezoelectric constant and leading to a twofold increase in piezoelectricity compared to pristine PLLA.

**Figure 6:**
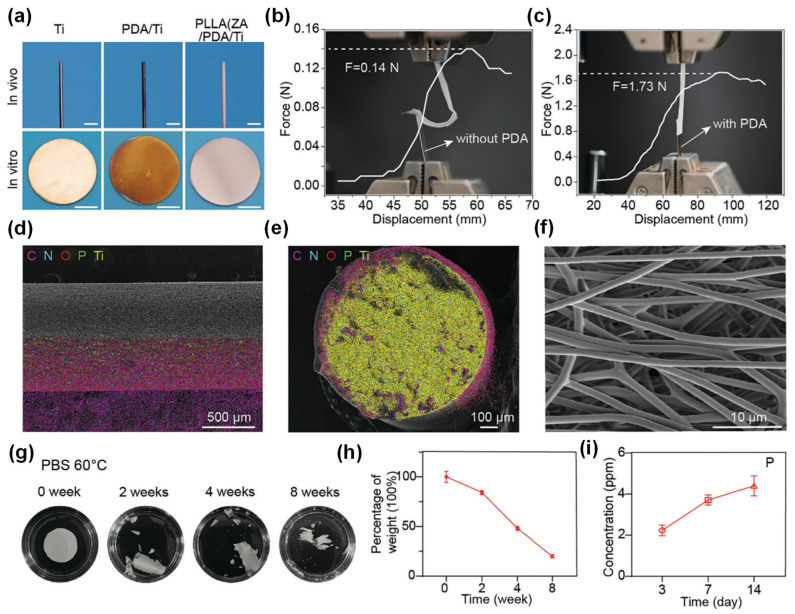
Preparation and characterization of piezoelectric PLLA/ZA coatings: (**a**) images of the preparation process of the titanium (Ti) surface coating. A layer of PDA was modified on the Ti surface first, and then PLLA/ZA coating was spun on the PDA. (**b**,**c**) Force–displacement curves required for the coating to be stripped from the Ti surface with or without PDA. (**d**) Side view and (**e**) cross-section SEM images of Ti rods with PLLA/ZA coating and the mapping distribution of the elements C, N, O, P, Ti, showing that ZA is uniformly dispersed in PLLA. (**f**) Representative SEM image of PLLA/ZA coating. (**g**) Images of the accelerated degradation process of PLLA/ZA coating and mass-time curve. (**h**,**i**) Test of ZA release rate represented by the element P. Reprinted with permission from ref. [68]. Copyright 2024, Wiley-CH.

Additive-based treatment has also been explored in advanced fabrication techniques like 3D printing, creating superior piezoelectric structures [67]. A notable example is the addition of zoledronic acid (ZA) to PLLA as a nucleating agent to boost the piezoelectric output. As shown in Figure 6, a study on PLLA/ZA composites [68] not only enhanced bone differentiation through the porous piezoelectric matrix but also inhibited osteoclast activity via the slow release of ZA, providing an effective method for preventing the aseptic loosening of bone implants. Long-term measurements demonstrated that the material decomposed over time, with the film showing signs of degradation within two weeks and an almost complete degradation within eight weeks.

### 4.2. Modifications in Processing Techniques

As mentioned, in polymers, the drawing process towards oriented chains is commonly employed to improve piezoelectric responses. This approach works well for amorphous or low-crystalline polymers because applying stress on large crystalline regions would lead to fracture. It is often necessary for drawn samples to undergo an annealing treatment to optimize their crystallinity. However, this process can also cause the polymer chains to no longer be oriented, since isotropic crystalline structures may form after annealing. Supercritical CO_2_ treatment can be an alternative way to achieve high piezoelectricity, as it could induce a higher-order crystalline structure within the polymer and transform a spherulitic structure into nano-rod-like crystals. Furthermore, it was demonstrated that supercritical CO_2_ treatment of drawn PLLA resulted in a doubling of the piezoelectric constant *d*_14_ [69].

### 4.3. Nanostructuring

In piezoelectric biopolymers, nanostructuring has become a popular approach for enhancing piezoelectric performance [8]. Reducing the physical dimensions of materials could be used to change their properties. Nanostructures, such as nanowires and nanotubes, have at least one dimension that is below 500 nm [70]. Molecular anisotropy is critical when working with small-scale nanostructured piezoelectric biopolymers because the drawing treatment cannot be conducted in such a situation. Notably, the growth process may induce a molecular alignment by itself [71].

**Figure 7:**
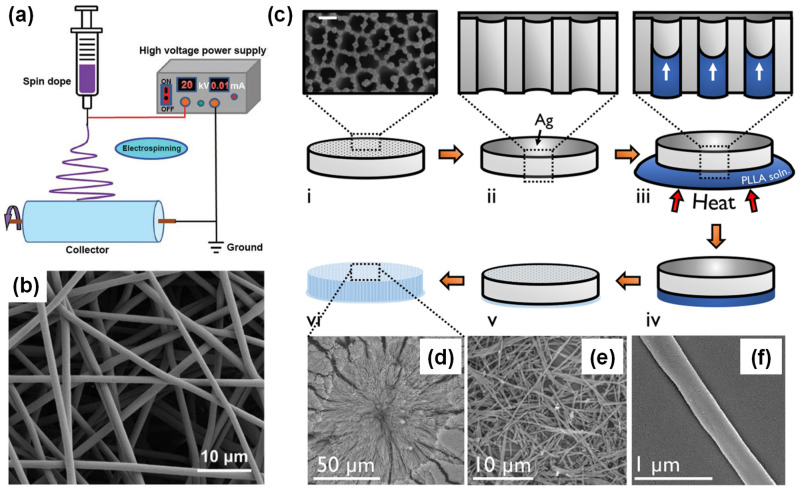
(**a**) Schematic of the electrospinning process. Reprinted with permission from ref. [74]. Copyright 2016, ELSEVIER. (**b**) Typical polymer nanofibers produced via electrospinning. Reprinted with permission from ref. [76]. Copyright 2019, ELSEVIER. (**c**) A schematic of the solution template wetting process used to grow PLLA nanowires: (**i**) The porous AAO template. (**ii**) A capping layer of silver is sputtered onto the uppermost surface of the template, sealing the pores from one side. (**iii**) The capped template is floated onto a droplet of 10 wt. % solution of PLLA in 1,4-dioxane. (**iv**) The infiltrated template is lifted from the droplet after 10 min. (**v**) Removing residual solvent. (**vi**) The template material is selectively etched in phosphoric acid for revealing the PLLA nanowires (**d**), (**e**) collected as a nanowire powder, and (**f**) an individual PLLA nanowire. Reprinted with permission from ref. [78]. Copyright 2017, AIP Publishing.

Electrospinning is a widely used technique for producing nanostructured polymer fibers (see in Figure 7a) [72,73,74] in which the polymer solution is processed under a strong electric field, overcoming the surface tension and producing a fine liquid jet. As the solvent evaporates, the wet polymer forms fibers with diameters ranging from tens to hundreds of nanometers. The flow-induced shear force during electrospinning can make the polymer chains oriented, replacing mechanical drawing. Additionally, the applied electric field can also impact the piezoelectric output by affecting the material’s properties [71].

The collection of electrospun polymer fibers is vital for achieving ideal piezoelectricity [75,76]. Random nanofibers produced by electrospinning may make the structure more centrosymmetric, which negatively impacts piezoelectricity. To avoid this, rotating collectors can help to have the fibers uniaxially aligned, creating a higher anisotropy and piezoelectric behavior of materials. Template wetting can be an effective technique for creating nanostructured biopolymers [77,78]. In this process, nanoporous templates are filled with polymer solutions or melts and formed into a solid state. Anodized aluminum oxide (AAO) can act as a template due to its chemical stability and customizable geometry. After removing the template, nanostructures like nanowires or nanotubes then occur during the infiltration process from i to iv in Figure 7c–f. The confined effects of nanopores induce a molecular alignment, suggesting that template wetting is a viable method for producing piezoelectric biopolymer nanostructures.

## 5. Applications in the Energy Harvesting and Biomedicine Fields

Electronic devices that integrate biomaterials can be developed in the field of biosensors, self-powered devices, electronic signal detectors, and others [79,80]. There have been plenty of instances of such devices based on piezoelectric biopolymers, advancing the development of an environmentally friendly, sustainable, and intelligent future [81].

### 5.1. Energy Harvesting

**Figure 8:**
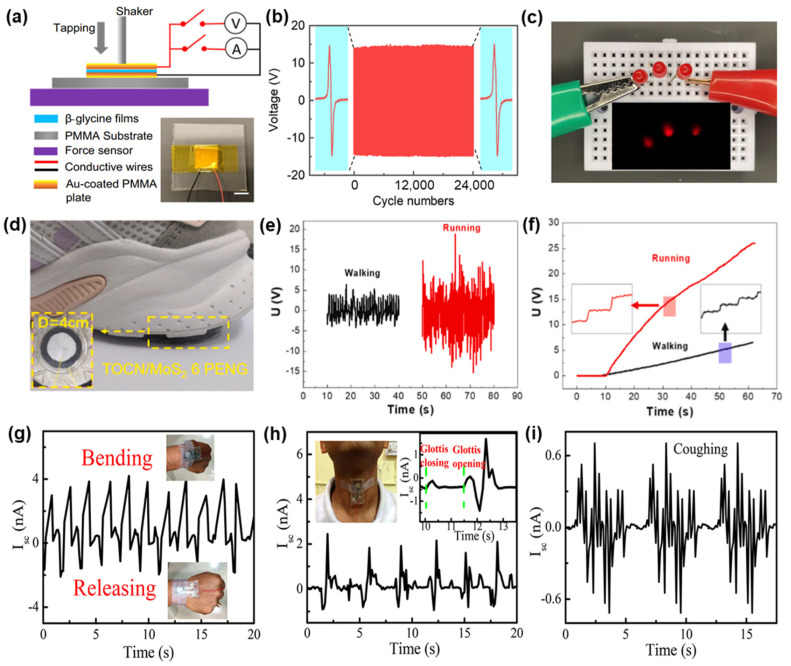
Piezoelectric electrical performance and mechanical stability of β-glycine nanocrystalline films: (**a**) schematic of the photography of a real device and piezoelectric measurement set-up. (**b**) Voltage output signals during 24,000 cycles under 1.5 MPa compressive pressure. (**c**) Image showing three LEDs lit up by an individual piezoelectric device. Reprinted with permission from ref. [82]. Copyright 2023, Springer Nature. Instantaneous output power corresponding to the (**d**) photograph of the TOCN/MoS_2_ 6 PENG device mounted on a sports shoe sole, (**e**) output voltage signals, and (**f**) charging curves of the TOCN/MoS_2_ 6 PENG device for harvesting the energy of walking and running generated by a person of about 70 kg. Reprinted with permission from ref. [36]. Copyright 2022, ELSEVIER. Time-dependent current responses during (**g**) repeated bending−releasing cycles of the wrist, (**h**) swallowing motions, (**i**) repeated coughing actions. Reprinted with permission from ref. [86]. Copyright 2017, ACS Publishing.

Piezoelectric biopolymers are greatly suitable for coupling mechanical energy and electrical energy in biological systems for the purposes of power generation. Applying them in piezoelectric nanogenerators (PENGs) to convert mechanical energy to electricity has made significant progress. β-Glycine films can exhibit increased performance with a piezoelectric voltage coefficient and thermostability of up to 192 °C before melting [82]. As shown in Figure 8a, a β-glycine film was prepared in sandwiched conductive polymethyl methacrylate (PMMA) substrates and measured with repeated force. The corresponding piezoelectric device exhibited an open-circuit voltage of 14.5 V and maintained a nearly constant output over 24,000 cycles. This considerable and stable output could enable a single device to light up three LEDs through finger tapping (see in Figure 8c), which is owed to the polar orientation and the compact crystalline structure of the β-glycine film.

Combining biopolymers with widely studied piezoelectric materials (e.g., ZnO, PVDF) can optimize the composite materials’ processing and produce energy conversion devices with superior performance [83]. There are numerous studies about hybrid cellulose films created by the assembly of vanadium-doped ZnO micro-flowers with bacterial cellulose (BC) which develop flexible PENGs and exhibit continuous piezoelectric signals in bending cycles, producing good values of output voltage and current density and possessing excellent mechanical robustness and durability. While PLLA fibers exhibit a lower piezoelectric response compared to PVDF in common configurations, PLLA outperforms PVDF in deconstructive configurations, which demonstrates that the output voltage and current follow a linear trend due to the well-aligned dipoles induced by the increased layers. As mentioned in Section 2.1, TOCN/MoS_2_ composite films show promise in manufacturing reliable PENGs with excellent mechanical robustness [84]. The corresponding devices could be integrated into shoes to collect power while moving (see Figure 8d,e) and could achieve an open-circuit voltage of 4.1 V and a short-circuit current of 0.21 μA, as well as charge a 10 μF capacitor to 1.6 V (see Figure 8f) [36]. By further incorporating BaTiO_3_ nanoparticles into the TOCN/MoS_2_ composite, higher outputs with an open-circuit voltage of 8.2 V and a short-circuit current of 0.48 μA were achieved [85]. These results reflect the significant capability and potential of piezoelectric biopolymers for energy harvesting.

Even with slight mechanical vibrations, electronic devices based on piezoelectric biopolymers can still monitor human physiological signals by harvesting the energy generated by tiny deformations. Self-assembled collagen nanofibers composed of fish skin can achieve electrostriction without electrical polarization [86]. The corresponding nanogenerator, called a pressure sensor, can generate an open-circuit voltage of 2 V and a short-circuit current of 20 nA under 1.8 MPa of applied pressure, and it exhibits an extremely fast response (4.9 ms) and high durability (75,000 cycles). This work shows that biopolymer-based piezoelectric devices can fully achieve good responses like traditional high-performance piezoelectric materials. As seen in Figure 8g–i, when attached to a person’s wrist via tape, this biopiezoelectric device produces output signals due to the movements of human skin: repeated bending and release result in the generation of signals. It can also be used for sensing the closing and opening of the glottis during swallowing and coughing.

**Figure 9:**
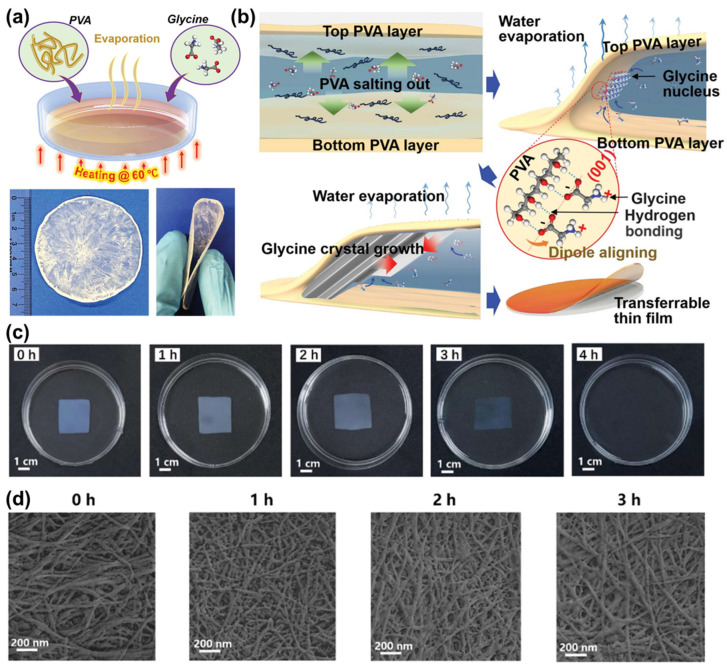
Piezoelectric glycine–PVA films: (**a**) schematic synthesis approach over a large area. Bottom images are digital photographs of a wafer-sized as-grown film (left) and largely curved film showing flexibility (right). (**b**) Schematic crystallization process. The inset shows the orientation alignment of glycine molecules at the PVA surface during nucleation, leading to long-range crystal alignment. (**c**) Piezoelectric voltage output at a 2:1 ratio measured under a 30 N impulse force. Reprinted with permission from ref. [87]. Copyright 2021, AAAS. (**c**) Optical and (**d**) SEM images demonstrate the degradation process of the sensor membrane, which is fully degraded in a cellulase solution. Reprinted with permission from ref. [89]. Copyright 2022, ACS Publishing.

Preparing piezoelectric films at a large scale generally remains an obstacle to their practical applications. However, this cannot be a barrier to the wide application of piezoelectric biopolymers. Wang et al. [87] proposed a large-scale fabrication method to obtain piezoelectric films. As shown in Figure 9a, they synthesized γ-glycine/polyvinyl alcohol (PVA) films by the direct solidification of mixed solutions. The solution was dispersed on the substrate surface and formed uniform liquid films due to the low surface tension. After the solvent evaporated, the liquid film crystallized from the edge and expanded rapidly within 30 min. γ-Glycine was first nucleated on the top surface near the edge of the liquid and then grew into the concentrated liquid between the PVA layers and crystallized into solid crystalline films, which was attributed to the sequential precipitation of these two materials. Following water evaporation, non-soluble PVA would precipitate with its amphiphilicity and aggregate at the interface, and the increasing concentration could activate the salting out of PVA, creating a concentration gradient from the top surface to the bulk solution (Figure 9b). These uniform and flexible PVA films with a sandwich structure, with γ-glycine self-assembling between the two layers, were all made with a 7 cm dimension scale and could easily move off the substrates.

The degradability of biopolymers makes them promising candidate materials for state-of-the-art recyclable electronic devices [88]. It is an advantage of biopolymers and also what makes them different from traditional piezoelectric materials. In addition to large-scale manufacturing, green manufacturing and post-processing are also topics of concern. A lot of piezoelectric polymers, like peptides, proteins, viruses, cellulose, chitin, PLLA, PHBV, etc., provide superior biocompatibility, biodegradability, and flexibility and have excellent advantages in environmental sustainability. Piezoelectric devices fabricated by composting bacterial cellulose (BC) hydrogel and imidazolium perchlorate (ImClO_4_) exhibit high sensitivity and a wide operating range of 0.2 to 31.25 kPa. Remarkably, this BC/ImClO_4_ film obtains excellent degradability. From the optical images and SEM images showing the biodegradation process (see Figure 9c,d) [89], the BC/ImClO_4_ film gradually degrades over time in the cellulase solution (5 mg/mL, 50 °C) and finally disappears after 4 h, and the fibrillar morphology of the film evolves from compact to rough.

Moreover, it is of great significance to combine biopolymers with biodegradable encapsulation materials (such as PLA, PCL, and PLGA) and bioabsorbable electrodes to explore fully degradable devices, thereby achieving the complete biodegradability of electronic materials and devices. This could be achieved by biopolymers and may become a new hot spot for future research on wearable electronic devices [90].

### 5.2. Biomedical Applications

Through innovative material preparation and structural design, biopiezoelectric materials will show great potential in the fields of medical health and body monitoring. As an amino acid-based piezoelectric material, glycine has been widely studied due to its polar structure, which contains an amino group and a carboxylic acid group, showing a high piezoelectric coefficient and excellent stability. Glycine can form a variety of crystalline phases, among which the β and γ phases have non-centrosymmetric structures that possess piezoelectric properties [91]. In addition, researchers have discovered some natural piezoelectric materials from animals, such as the fish skin collagen mentioned above. Through the electrospinning technique, collagen nanofibers have been successfully prepared and are used in electronic skin to monitor human health. These findings further enrich the types and application fields of piezoelectric biomaterials [10].

Piezoelectric biopolymers can also be employed in fabricating biomedical sensors and other products [92,93,94]. Tajitsu et al. [95] fabricated a smart medical tweezer and catheter via high-speed spinning using electrospun PLLA fibers to promote piezoelectricity. Then, they deposited electrodes onto the fibers to make an actuator that can sense an applied alternating current voltage. This actuator functions as a medical tool, demonstrating its potential for use in procedures such as removing thrombosis samples. The degradation tests showed that the PLLA fibers began to decompose after 4 weeks, and they were completely degraded after 12 weeks, demonstrating their biodegradability under a biological environment [96,97]. Due to their eco-friendliness and scalability in PENGs and biosensors [98], Yang et al. [99] fabricated PLLA/ZnO composite fibers without post-processing techniques like polarization. The oriented fibers showed an enhanced crystallinity and piezoelectric performance with an open-circuit voltage of 7.9 V and a short-circuit current of 286 nA, which are about several times higher than those of random samples, sufficient for charging a 10 μF capacitor to 2 V in 210 s and powering an LED. Furthermore, the high flexibility and sensitivity of this device make it excellent for detecting human motion and monitoring health conditions, like coughing and pulsing.

**Figure 10:**
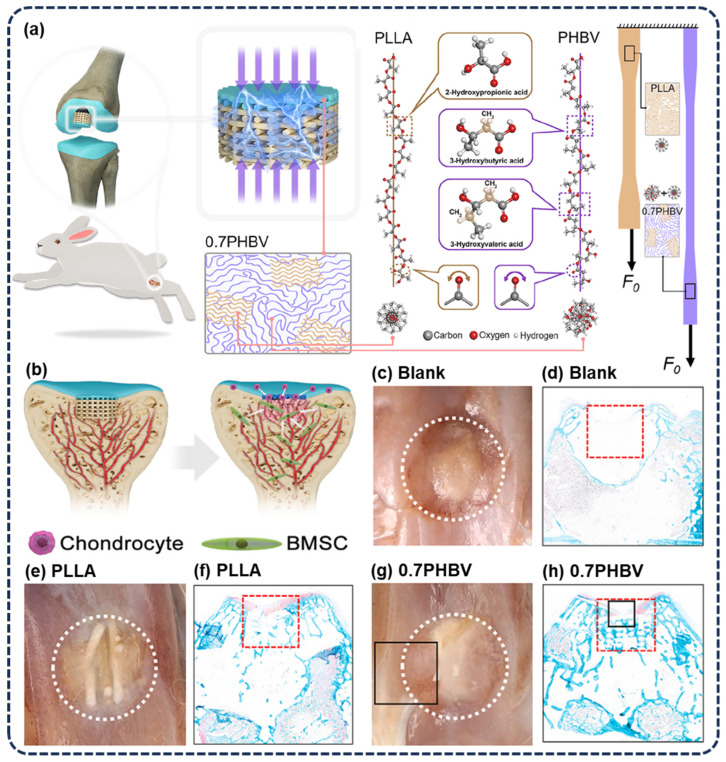
Schematic diagram of (**a**) 3D printing piezoelectric materials. (**b**) A piezoelectric scaffold was implanted into the defect zones of the trochlear groove of a rabbit’s joint to modulate cartilage remodeling. (**c**–**h**) Macrophotography confirmed the piezoelectric scaffold of 0.7PHBV was well fused and connected with the adjacent tissue at 18 weeks post-operation. Compared to the control group’s histological staining, both piezo groups of PLLA and 0.7PHBV presented superior cartilage restoration. Reprinted with permission from ref. [100]. Copyright 2024, ELSEVIER.

It has been shown that PLLA can be used for tissue repair, but its low piezoelectric coefficient limits its potential for application in in situ piezoelectric stimulation therapy. Wang et al. [100] developed a biodegradable piezoelectric composite composed of PLLA and PHBV (see Figure 10a) whose piezoelectric coefficient is 2.0 to 2.5 times that of pure PLLA. This composite material achieves partial miscibility and higher piezoelectric performance by combining two homologous monomers with similar chiral carbon atoms and C=O dipoles. The study found that this kind of composite biopolymer can effectively promote cartilage repair in vivo. Experiments in rabbit cartilage defects showed that the bio-piezoelectric scaffold significantly enhanced the remodeling ability of cartilage (see Figure 10c–g). These findings can be helpful for developing piezoelectric biopolymers with low modulus, good processing performance, and excellent biodegradability, showing potential applications in regenerative medicine, especially in articular cartilage repair. Meanwhile, this also shifts the focus of bio-piezoelectric material applications to regenerative medicine.

**Figure 11:**
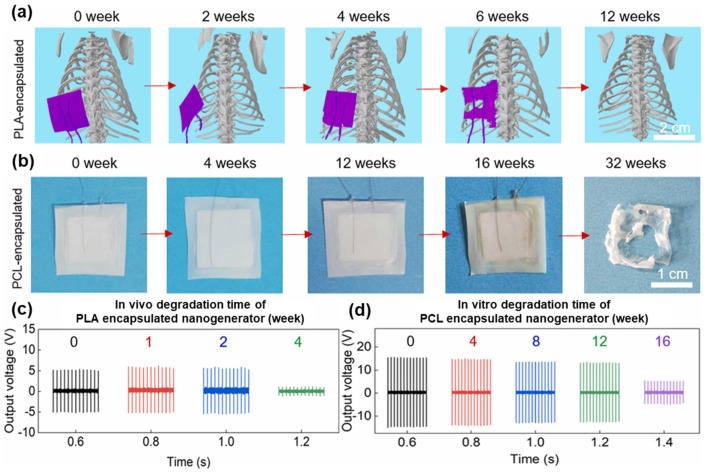
The biocompatibility and biodegradability of PHBV/PLLA/KNN PENG: (**a**) micro-CT images of the PLA-encapsulated PHBV/PLLA/KNN PENG. (**b**) Biodegradation performance of the PCL-encapsulated PHBV/PLLA/KNN PENG at 32 weeks. Voltage outputs of the (**c**) PLA- and (**d**) PCL-encapsulated PENG measured at different times (ultrasound frequency 100 kHz, ultrasonic intensity 0.3 W/cm^2^). Reprinted with permission from ref. [101]. Copyright 2022, ELSEVIER.

Completely degradable implantable devices have high prospects for medical applications. One study proposed a method for preparing a completely biodegradable PENG for in vivo electrical stimulation (ES) to repair peripheral nerve damage while avoiding a second surgery to remove the implanted device out of the body [101]. The PENG consisted of piezoelectric biopolymers (PHBV, PLLA, KNN) and a PLA or poly-ε-caprolactone (PCL) encapsulation layer, as well as magnesium electrodes and molybdenum wires, which are all biodegradable. With the help of ultrasound, this implanted PENG delivered tuned ES to the nerve conduction catheter, greatly enhancing nerve regeneration. In biodegradability tests comprising immersion in PBS solution at 37 °C, the PLA-encapsulated PENG could produce stable output in vivo for at least 2 weeks but could no longer be detected under micro-CT (Figure 11a) after 12 weeks, indicating that it had been completely biodegraded. The PCL-encapsulated device maintained good structural integrity and 80% output performance of its initial value after 12 weeks (Figure 11c,d). Obvious degradation and a complete disappearance of this device happened after 32 weeks, indicating that the PCL-encapsulated PENG can be used for long-term and stable ES in vivo for more than 12 weeks. The work provides a new strategy for using biodegradable PENGs for tissue engineering applications.

## 6. Conclusions

Piezoelectric biopolymers possess biocompatibility, biodegradability, and flexibility, demonstrating great potential in advancing technologies in bioelectronics, energy harvesting, and biomedicine; this makes them excellent candidates for next-generation devices and environmentally friendly applications. The developments in biopolymer-based nanogenerators have verified the success of self-powered bioelectronic devices. More efforts in processing methodologies have significantly enhanced the molecular orientation and crystallinity of polymers and improved their piezoelectric performance. The integration of additives, fillers, and structure tuning has also provided more options for promoting the output of piezoelectric biopolymers, with applications in energy harvesting and sensor technology.

Several critical challenges remain in optimizing the performance of biodegradable piezoelectric polymers, such as their relatively low output and low stability. Thus, it is important to enhance the piezoelectric response through molecular engineering, crystallization adjusting, and effective fabrication methods. Additionally, research studying the stability, degradation behavior, and biocompatibility of these materials in open-air environments needs more effort. These polymers can be widely used in biomedical and wearable devices. A comprehensive understanding of their interactions with biological tissues and their degradation under various conditions is critical to explore their long-term functionality and safety. Piezoelectric biopolymers represent desirable developments for a sustainable future. Continued efforts will help these eco-friendly biopolymer materials become foundational components in the next generation of biocompatible, self-powered, and sustainable electronic devices, creating new opportunities for technological advancement.

## Data Availability

The original contributions presented in the study are included in the article, further inquiries can be directed to the corresponding author.
